# Percutaneous Coronary Intervention in Africa: A Systematic Review of Associated Outcomes

**DOI:** 10.7759/cureus.88488

**Published:** 2025-07-22

**Authors:** Chibuike F Obi, Collins C Okeke, Augusta U Onyema, Abdulahi Zubair, Shivangi Sharma, Chimaobi O Nwevo, Faramoluwa E Akinyanmi, Emmanuel O Okwor, Nzubechukwu S Ozokwelu, Temitope Akinnuoye

**Affiliations:** 1 General Internal Medicine, Fairfield General Hospital - Northern Care Alliance NHS Foundation Trust, Manchester, GBR; 2 Internal Medicine, University of Port Harcourt Teaching Hospital, Port Harcourt, NGA; 3 General Practice, Pennine North West GP Training Scheme, Manchester, GBR; 4 Neurosurgery, Surgery Interest Group of Africa, Lagos, NGA; 5 Internal Medicine, South Brooklyn Health, New York City, USA; 6 Internal Medicine, Sacred Heart Hospital, Abeokuta, NGA; 7 Public Health, YOHAN Research Institute, Enugu, NGA; 8 Gastroenterology, Fairfield General Hospital - Northern Care Alliance NHS Foundation Trust, Manchester, GBR; 9 Internal Medicine, Fairfield General Hospital - Northern Care Alliance NHS Foundation Trust, Manchester, GBR

**Keywords:** acute coronary syndrome, africa, coronary artery disease, outcomes, percutaneous coronary intervention

## Abstract

Percutaneous coronary intervention (PCI) is a minimally invasive procedure that plays an important role in relieving an occlusion of the coronary arteries, allowing blood circulation to the cardiac tissues. It is central to the management of coronary heart disease. In recent years, there has been an increase in the use of PCI across the African continent, and this review aims to evaluate and report the clinical outcomes of PCI use in Africa among patients with coronary heart disease. A comprehensive search was conducted on PubMed, EMBASE via Ovid, and AJOL (African Journals Online) from inception to February 2025. The Preferred Reporting Items for Systematic Reviews and Meta-Analyses (PRISMA) guideline was used in this study. Three hundred two articles underwent full screening following predefined eligibility criteria, and 31 articles were included for qualitative analysis. The primary outcomes assessed were all-cause mortality and major adverse cardiovascular events (MACE). Secondary outcomes included post-procedural outcomes like complications and post-procedure thrombolysis in myocardial infarction (TIMI) flow grade. This review synthesized 31 articles from nine African countries. A total of 11,507 patients participated in this study, and 10,701 patients underwent PCI procedures, with stenting being the most common technique employed. Among participants, 8,620 (74.3%) are male, and 2,887 (24.8%) are female. A total of 170 (17.8%) cases of MACE were reported, and an overall mortality of 457 (4.9%) was reported. In-hospital mortality accounted for 317 of the total mortality, making up 4.7% of the assessed patients. About 78.2% of patients who underwent PCI achieved post-procedure TIMI flow grade III, an indicator of post-procedure reperfusion success rate in the study. The most common complications reported include: Heart failure (19.1%), arrhythmia (9.1%), revascularization (8.9%), coronary events (8.1%), structural complications (8.1%), and the need for intensive care unit support (7.9%). An allergic reaction (4.7%), a rare complication, was also reported. The study shows that mortality rates are relatively higher when compared to higher-resource countries. STEMI remains the predominant indication for PCI, underscoring a healthcare system that is still largely reactive rather than preventive. The high burden of comorbidities such as smoking, hypertension, and diabetes, paired with complication rates including heart failure and MACE, reflects systemic gaps in both acute and chronic cardiovascular care. To improve outcomes and close the equity gap, African health systems must prioritize context-sensitive guideline implementation, capacity building, and continuity of care.

## Introduction and background

Percutaneous coronary intervention (PCI) (also known as coronary angioplasty) is one of the most commonly performed medical procedures in the world and was first successfully performed by Andreas Gruntzig in 1977 [1]. It’s a minimally invasive procedure that relieves occluded coronary arteries and improves the blood supply to the affected cardiac tissue [2]. It is indicated in emergency presentations with acute coronary syndrome (ACS) or as an elective procedure in cases of stable ischemic heart disease. PCI can also be used for critical coronary artery stenosis that does not qualify for coronary artery bypass graft (CABG) [2].

Coronary artery disease (CAD) is a disease process characterized by the build-up of atherosclerotic plaque within the lumen of coronary arteries, leading to narrowing or occlusion of these arteries and consequent reduction of blood supply to their relevant cardiac tissue. Often used interchangeably with coronary heart disease (CHD), CAD encompasses conditions like ACS, stable angina, and silent myocardial ischemia [3].

Recent global reports indicate cardiovascular disease as the leading cause of global burden of disease, accounting for 9.44 million deaths and 185 million disability-adjusted life years (DALYs) with an estimated global prevalence of about 1.74% [4]. In Africa, cardiovascular diseases contribute to over a third of noncommunicable disease deaths, with 22.9 million DALYs [5]. Between 1990 and 2020, Africa recorded an almost 50% increase in the prevalence of cardiovascular diseases and a doubling of deaths from ischemic heart diseases [6]. Within this time, cardiovascular disease has climbed from the 6th to the 2nd leading cause of death in sub-Saharan Africa [5]. This astronomical rise in the prevalence of CAD can be attributed to an increase in its well-established risk factors. These include hypertension, dyslipidemia, obesity, diabetes, tobacco smoking, alcohol misuse, sedentary lifestyle, and socioeconomic factors like urbanization and nutritional changes (consumption of highly processed food), which have equally been noted to be major contributors to the development of this epidemiological trend [4,5,7,8].

Despite the increasing cases of CAD in Africa, there are notable limitations to its management on the continent. One of the major challenges is the limited availability of PCI services in many African countries [7]. Available data suggest that there is a shortage of cardiac catheterization labs in most African countries, except for Tunisia and Egypt [9].

Notwithstanding, many African countries have witnessed a proliferation of PCI labs in recent years [10-12], although, unlike in developed and high-income countries, there is limited data and evidence reporting outcomes of this procedure in Africa. Given the increase in PCI procedures on the continent, studying its associated outcomes is important to establish the effectiveness of the procedure in Africa. This study aims to review available literature and report on the associated clinical outcomes of PCI in patients with coronary heart disease (stable angina, ACS, and silent myocardial ischemia) in Africa.

## Review

Methods

This review was conducted using the preferred reporting system for systematic review and meta-analysis (PRISMA) guidelines [13]. The study protocol was registered with the International Prospective Register of Systematic Reviews (PROSPERO) - CRD420251062965.

The eligibility criteria used are displayed in Table 1.

**Table 1:** Eligibility Criteria NSTEMI: Non-ST elevation Myocardial Infarction; STEMI: ST elevation myocardial infarction; MACE: major adverse cardiovascular event; PCI: percutaneous coronary intervention

Inclusion Criteria	Exclusion Criteria
Original peer-reviewed papers	Abstracts, case reports, case series, systematic reviews, narrative reviews, meta-analyses
Adult patients (>/=18 years) of any gender	Patients < 18 years
Studies on patients who have undergone percutaneous coronary intervention as a treatment for coronary heart disease (stable angina, unstable angina, NSTEMI, STEMI, or silent myocardial infarction)	Non-English articles
Studies done in any African country	Non-African studies
Studies that reported at least one clinical outcome of interest (Mortality, MACE, complications, or success rate).	Studies that did not report any outcome of interest
	Articles with a sample population of coronary heart disease patients treated with different modalities, including PCI, but did not report outcomes specific to those treated with PCI.

Search Strategy

A comprehensive search was carried out on PUBMED, EMBASE via Ovid, and AJOL (African Journals Online) from inception to the 12th of February 2025. Using Boolean word operators “OR” and “AND”, a combination of key words and Medical Subject Headings (MeSH) terms relating to Coronary Intervention (“Percutaneous Coronary Intervention” OR “PCI” OR “Coronary reperfusion” OR “Coronary angioplasty” OR “Coronary revascularization” OR “drug eluting stent” OR “stent” OR “Angioplasty” OR “drug-eluting balloon” OR “percutaneous transluminal coronary angioplasty”), treatment outcomes (“Treatment Outcome” OR “Outcomes” OR “Mortality” OR “major adverse cardiovascular event” OR “MACE” OR “Complications” OR “Length of hospital stay” OR “Success rate”) and Africa (all African countries listed individually) were employed in developing the search strategy to ensure all relevant studies were captured. The search strings used are fully displayed in the Appendices section (Table 9).

Data Selection

The search results were imported to Rayaan referencing software (a systematic review software; Rayyan Systems, Cambridge, MA) for removal of duplicates and detailed screening [14].

After duplicate removal, the remaining articles were independently screened by two reviewers according to the eligibility criteria. Initial screening was based on their title and abstracts. This was followed by a full-text review of the remaining articles to select the articles eligible for this study. Any conflict in opinion was resolved by a consensus between all authors.

Data Extraction and Outcomes of Interest

Data was extracted from the eligible studies by two reviewers and entered into a pre-formatted Microsoft Excel spreadsheet. Disagreements were discussed among reviewers; in the case of no resolution, an appeal was made to another reviewer. The extracted baseline variables included author name, country and year of publication, sample size, gender, mean age, patient risk factors (hypertension, diabetes mellitus, dyslipidemia, history of CAD, smoking), PCI procedural characteristics (PCI technique used, type of stents - drug eluting vs bare-metal stent, occluded vessels, femoral/radial access), follow-up time.

Data related to the outcomes were equally extracted: Overall all-cause mortality, in-hospital mortality, major adverse cardiovascular events (MACE), post-procedure thrombolysis in myocardial infarction (TIMI) flow grade, and post-procedure complications.

The primary outcomes of interest were all-cause mortality and MACE. MACE was extracted as defined in the individual studies. Secondary outcomes included post-procedural outcomes like complications and post-procedure TIMI flow grade, which is an indicator of post-procedure reperfusion success [15] and a strong predictor of mortality outcomes following the procedure [16].

Quality Assessment

The included articles underwent quality assessment using the Joanna Briggs Institute (JBI) risk of bias critical appraisal tool for cohort, cross-sectional, and randomized control trials [17-19]. The purpose of this appraisal is to assess the methodological quality of a study and to determine the extent to which a study has addressed the possibility of bias in its design, conduct, and analysis. Articles are assessed with a yes, no, not clear, and not applicable. This was done by two reviewers.

Results

**Figure 1:**
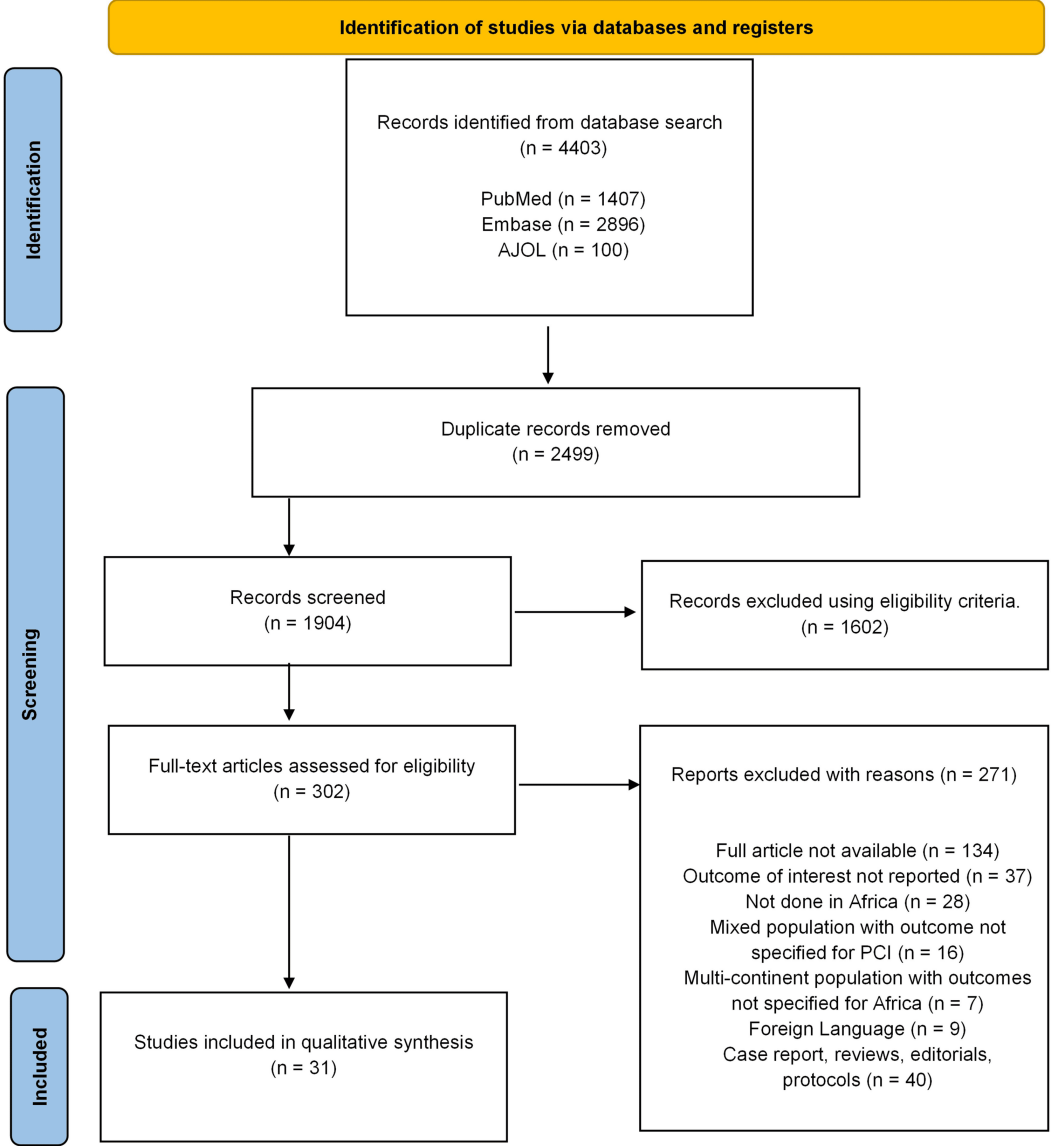
PRISMA flow diagram AJOL: African Journals Online, PCI: percutaneous coronary intervention

Our search generated 4,403 articles in the first instance, but 2,499 articles were removed following deduplication. In total, 1,904 articles underwent title and abstract screening following the eligibility criteria, leading to the removal of a further 1,602 articles after this round of screening. Three hundred and two (302) articles underwent full-text screening, 271 articles were removed following full-text screening due to several factors, including the unavailability of the full article, no reported outcomes, research not conducted in Africa, the article being non-English, a multi-continental population, and outcomes not specific to African patients. Thirty-one articles were included for the final quantitative analysis. It is pertinent to note that protocols, reviews, case reports, and editorials were excluded from this review. The PRISMA chart summarizing the screening process is presented in Figure 1.

Study Characteristics

**Figure 2:**
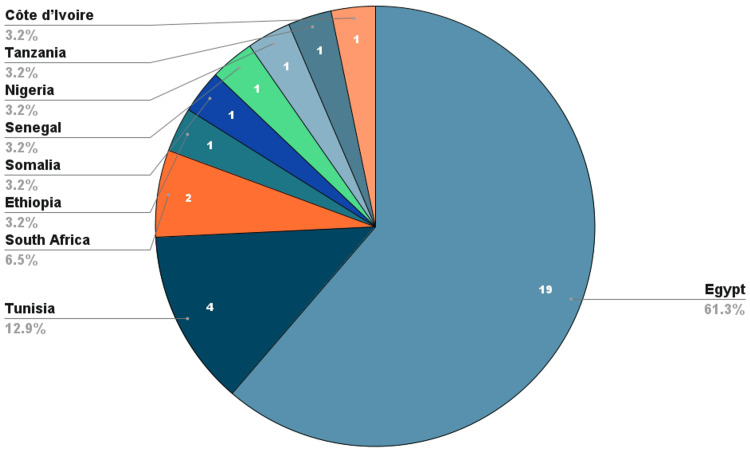
Countries of study

All the studies in this review came from nine (9) African countries (Egypt, South Africa, Tunisia, Ethiopia, Somalia, Senegal, Nigeria, Tanzania, and Côte d’Ivoire) with Egypt contributing the most with nineteen (19) papers, followed by Tunisia with four (4), South Africa with two (2) and the other countries contributed one (1) paper each as shown in Figure 2. The study period ranged from 2008 to 2024.

Of the studies included in this paper, seventeen studies are cohort studies [20-36], six studies are cross-sectional [37-42], and eight studies are randomized controlled trials [43-50].

Demographical Characteristics

From the 31 included studies, there were a total of 11,507 participants, with 8,620 male participants representing (74.3%) of all participants, and 2,887 female participants (24.9%), with one study not including the gender distribution of their sample size [50]. The study with the largest sample size had 3,627 patients, while 30 was the smallest sample size.

The mean age ranged from 53 to 66 years, but was absent in two studies [34,50]. Of these, 10,701 patients received PCI, and the follow-up period ranged from 1 to 24 months. The follow-up period was absent in 12 studies [20,25,27,34,35,37,40-42,44,46,49]. The full details of the study characteristics are shown in Table 2.

**Table 2:** Study characteristics

Author	Year	Country	Sample Size	Patients With PCI	Mean Age	Male	Female	Follow Up (Months)
Abdo et al. [20]	2024	Egypt	500	500	58	281	219	N/A
Araquib et al. [21]	2024	Egypt	72	72	53	62	10	6
Khalil et al. [22]	2021	Egypt	123	123	54	95	28	6
Rekik et al. [23]	2009	Tunisia	81	81	56	73	8	6
Salem et al. [24]	2015	Egypt	40	40	56	32	8	1
Shalaby et al. [25]	2024	Egypt	1187	1187	66	920	267	N/A
Shashu and Baru [26]	2022	Ethiopia	197	197	58	159	38	1
Sobhy et al. [27]	2012	Egypt	1324	835	56	993	331	N/A
Zghal et al. [28]	2012	Tunisia	611	611	58	492	119	23
Amin and Alaarag [29]	2020	Egypt	303	303	57	211	92	7
Ben Abdessalem et al. [30]	2023	Tunisia	83	83	56	63	20	24
Kozuki et al. [31]	2010	South Africa	37	37	57	25	12	6
Mboup et al. [32]	2018	Senegal	110	110	60	95	15	1
Ghariani et al. [33]	2022	Tunisia	116	116	60	86	30	24
Hassan et al. [34]	2024	Egypt	3627	3627	N/A	2871	756	N/A
Hassan et al. [35]	2024	Somalia	103	80	58	75	28	N/A
Khalfallah et al. [36]	2020	Egypt	660	660	54	368	292	3
Ndaba et al. [37]	2023	South Africa	677	365	55	533	144	N/A
Refaat et al. [38]	2021	Egypt	400	400	56	284	116	6
Shawky et al. [39]	2023	Egypt	102	102	60	64	38	12
Johnson et al. [40]	2014	Nigeria	80	48	60	53	27	N/A
Ekou et al. [41]	2020	Côte d’Ivoire	166	166	54	152	14	N/A
Hooda et al. [42]	2023	Tanzania	227	227	62	185	42	N/A
Ali and Ahmed [43]	2024	Egypt	97	97	54	85	12	1
Al-Shaer et al. [44]	2022	Egypt	48	48	56	29	19	N/A
Mohamed-Ghazal et al. [45]	2024	Egypt	108	108	52	94	14	6
Shehata and Hamza [46]	2015	Egypt	130	130	55	67	63	N/A
Shehata et al. [47]	2015	Egypt	118	118	56	80	38	6
Tantawy et al. [48]	2021	Egypt	30	30	59	17	13	2 days
Imam et al. [49]	2024	Egypt	150	150	54	76	74	N/A
Nammour et al. [50]	2024	Egypt	100	50	N/A	N/A	N/A	12

Patient-Related Factors

**Figure 3:**
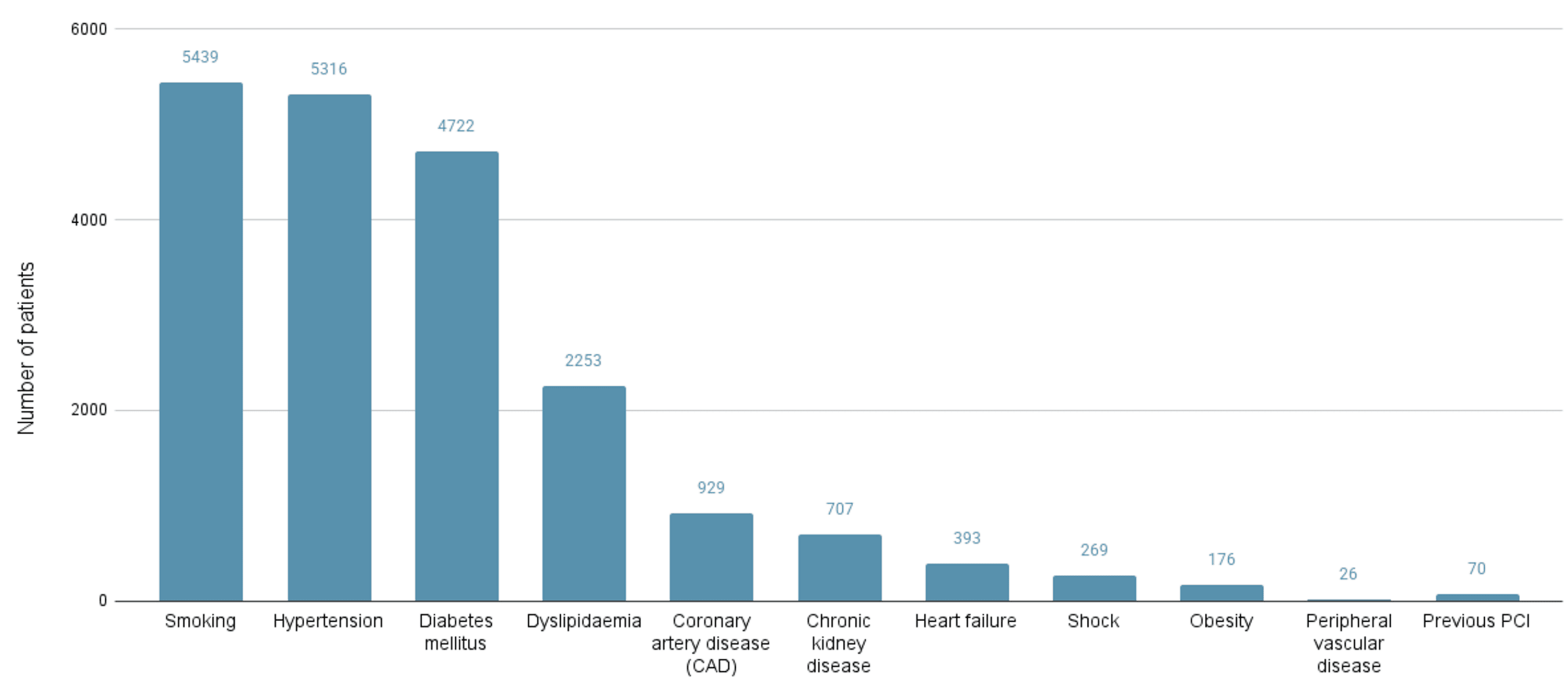
Patient-related risk factors CAD: coronary artery disease; PCI: percutaneous coronary intervention

Among all the comorbidities reported in the studies reviewed, the majority of patients (5439) were noted to be smokers, making smoking the most common reported comorbidity/risk factor. Other common reported patient-related comorbidity/risk factors included hypertension (5,316), diabetes mellitus (4,772), dyslipidemia (2253), family or personal history of CAD (929), chronic kidney disease (707), and heart failure (393). Figure 3 below shows the risk factors reported in the included study.

Outcome of Percutaneous Coronary Intervention

In the studies reviewed, 10,701 patients underwent PCI. Only eight articles reported on access sites and showed that the radial artery, used in 56.8% (2,670) of patients, was the most common site of access employed during this procedure. The pattern of vascular involvement was reported in 13 articles. 60.2% of the patients had an affected Left anterior descending artery (LAD), making it the most commonly affected vessel that required PCI. This was followed by the right coronary artery (30.8%). Some of the studies also reported patients having more than one affected vessel. Among the included articles, 16 articles reporting on the PCI method used, PCI with stent was the most common technique employed, and was used in 97% of cases. Drug-eluting stent (33%) and bare-metal stent (34.4%) showed a near-equal representation, although some studies that reported using stents did not specify the type of stent. A total of 25 articles assessed indications for PCI and showed STEMI to be the most reported reason for PCI, accounting for 90% of cases. Table 3 below summarizes the procedure characteristics.

**Table 3:** Procedure characteristics n - Number of patients with outcome, N - Total sample population of which a procedure characteristic was reported. NSTEMI: Non-ST elevation myocardial infarction; STEMI: ST elevation myocardial infarction; PCI: percutaneous coronary intervention

PCI characteristics	Number of patients (n/N)	Percentage (%)
Site of Access		
Femoral artery	2029/4699	43.2
Radial artery	2670/4699	56.8
Pattern of Coronary Artery Involvement		
Left anterior descending artery (LAD)	3445/5718	60.2
Right coronary artery (RCA)	1759/5718	30.8
Left circumflex artery (LCX)	1001/5718	17.5
Left main stem (LMS)	149/5718	2.6
Ramus	44/5718	0.8
Obtuse marginal artery (OMA)	19/5718	0.3
Posterior descending artery (PDA)	6/5718	0.1
PCI Techniques		
PCI with stents	3313/3414	97
Bare-metal stents	1175	
Drug-eluting stents	1125	
Balloon angioplasty	101/3414	3
PCI Indication		
STEMI	8503/9447	90.0
NSTEMI	363/9447	3.8
Unstable angina	201/9447	2.1
Stable angina	218/9447	2.3
Coronary artery disease	162/9447	1.7

Post-procedure reperfusion success was determined using the TIMI flow grading system [15]. As seen in Table 4, a greater number of patients who underwent PCI achieved a post-procedure TIMI flow grade III (78.2%), while a smaller number had a TIMI flow grade below III. Across the 10 studies that reported post-procedure TIMI flow grade, TIMI grade III was recorded in 78.2% of the patients - an indicator of the rate of success at achieving post-procedure reperfusion in this study.

**Table 4:** Post-procedure TIMI flow grade TIMI: Thrombolysis in myocardial infarction

TIMI Flow Grade	Number of Patients	Percentage (%)
0	67	3.7
I	111	6.1
II	216	12.0
III	1,413	78.2

There are lots of complications reported by various included articles, however the most reported complications are heart failure (19.1%), arrhythmia (9.1%), revascularization (8.9%), coronary events (8.1%), structural complications (8.1%), and the need for intensive care unit (ICU) support (7.9%). Other notable complications are hemodynamic shock (6.1%), contrast-induced nephropathy (5.9%), pulmonary edema (5.0%), and cardiac arrest (4.7%). An allergic reaction (4.7%), which is a rare complication, was observed in one study. MACE was reported in 12 studies, and a total of 170 (17.8%) cases were reported. A total of 457 (4.9%) mortalities were recorded among the 27 included articles, and in-hospital mortality accounted for 317 cases of the total mortality - representing 4.7% of patients across the 10 articles that reported in-hospital mortality. The full details of the post-procedure outcomes of PCI are shown in Table 5.

**Table 5:** Post-procedure Outcomes of PCI n - Number of patients with outcome, N - Total sample population from which the outcome was assessed MACE: major adverse cardiac event; TVR: target vessel revascularization; TLR: target lesion revascularization; AKI: acute kidney injury; LV: left ventricle; ICH: intracerebral hemorrhage; ICU: intensive care unit

Post-procedure Complication	Patients n/N	Percentage of Patients Affected (%)	No of Studies that Assessed the Outcome
Overall mortality	457/9,358	4.9	27
In-hospital mortality	317/6,757	4.7	10
MACE	170/954	17.8	12
Contrast-induced nephropathy (AKI)	296/5007	5.9	6
Revascularization (TLR & TVR)	96/1080	8.9	11
Stroke/ICH	39/5244	0.7	10
Pulmonary edema	59/1187	5.0	1
Heart failure	527/2751	19.1	9
Coronary event (Re-infarction/Angina)	200/2468	8.1	17
Bleeding/Hematoma	86/5106	1.7	11
Hemodynamic deterioration/Shock	135/2231	6.1	4
Cardiac arrest	87/1847	4.7	2
Structural complications (Ischemic cardiomyopathy, perforation, dissection, side branch occlusion)	40/493	8.1	4
Arrhythmia	83/910	9.1	5
Thrombotic event (Stent thrombosis/LV thrombus)	171/6130	2.8	9
Need for ICU support (Ventilation, vasopressors, hemodialysis)	111/1414	7.9	2
Allergic reaction	7/150	4.7	1

Tables 6-8 display the JBI critical appraisal tool for the included articles for cohort, cross-sectional, and randomized controlled trials.

**Table 6:** JBI critical appraisal tool for cohort studies. JBI: Joanna Briggs Institute

Checklist Questions	Abdo et al. [20]	Araquib et al. [21]	Khalil et al. [22]	Rekik et al. [23]	Salem et al. [24]	Shalaby et al. [25]	Shashu and Baru [26]	Sobhy et al. [27]	Zghal et al. [28]	Amin and Alaarag [29]	Ben Abdessalem et al. [30]	Kozuki et al. [31]	Mboup et al. [32]	Ghariani et al. [33]	Hassan et al. [34]	Hassan et al. [35]	Khalfallah et al. [36]
1. Were the two groups similar and recruited from the same population?	Y	Y	Y	Y	Y	Y	Y	Y	Y	Y	Y	Y	N/A	N/A	N/A	Y	Y
2. Were the exposures measured similarly to assign people to both exposed and unexposed groups?	Y	Y	Y	Y	Y	Y	Y	Y	Y	Y	Y	Y	N/A	Y	Y	Y	Y
3. Was the exposure measured in a valid and reliable way?	Y	Y	Y	Y	Y	Y	Y	Y	Y	Y	Y	Y	Y	Y	Y	Y	Y
4. Were confounding factors identified?	Y	Y	Y	Y	Y	Y	Y	Y	Y	Y	Y	Y	N	Y	Y	Y	Y
5. Were strategies to deal with confounding factors stated?	Y	Y	Y	Y	Y	Y	Y	Unclear	Y	Y	Y	N	N	Y	Y	Y	Y
6. Were the groups/participants free of the outcome at the start of the study (or at the moment of exposure)?	Y	Y	Y	Y	Y	Y	Y	Y	Y	Y	Y	Y	Y	Y	Y	Y	Y
7. Were the outcomes measured in a valid and reliable way?	Y	Y	Y	Y	Y	Y	Y	Y	Y	Y	Y	Y	Y	Y	Y	Y	Y
8. Was the follow-up time reported and sufficient to be long enough for outcomes to occur?	Y	Y	Y	Y	Y	Y	Unclear	Y	Y	Y	Y	Y	Y	Y	Y	Y	Y
9. Was follow-up complete, and if not, were the reasons for the loss to follow-up described and explored?	Y	N	Y	Y	Y	Y	Y	Y	Y	Y	N	Y	Y	Y	Y	Y	Y
10. Were strategies to address incomplete follow-up utilized?	N	N	N/A	N/A	N/A	N/A	N/A	N/A	N/A	N/A	N	N/A	N/A	N/A	N/A	N/A	N/A
11. Was appropriate statistical analysis used?	Y	Y	Y	Y	Y	Y	Y	Y	Y	Y	Y	Y	Y	Y	Y	Y	Y

**Table 7:** JBI critical appraisal tool for cross-sectional studies. JBI: Joanna Briggs Institute

Checklist Questions	Ndaba et al. [37]	Refaat et al. [38]	Shawky et al. [39]	Johnson et al. [40]	Ekou et al. [41]	Hooda et al. [42]
1. Were the criteria for inclusion in the sample clearly defined?	Y	Y	Y	Y	Y	Y
2. Were the study subjects and the setting described in detail?	Y	Y	Y	Y	Y	Y
3. Was the exposure measured in a valid and reliable way?	Y	Y	Y	Y	Y	Y
4. Were objective, standard criteria used for measurement of the condition?	Y	Y	Y	Y	Y	Y
5. Were confounding factors identified?	Y	Y	Y	N	N	Y
6. Were strategies to deal with confounding factors stated?	Y	Y	Y	N	N	Y
7. Were the outcomes measured in a valid and reliable way?	Y	Y	Y	Y	Y	Y
8. Was appropriate statistical analysis used?	Y	Y	Y	Y	Y	Y

**Table 8:** JBI critical appraisal tool for randomized control studies. JBI: Joanna Briggs Institute; RCT: randomized controlled trial

Checklist Questions	Ali and Ahmed [43]	Al-Shaer et al. [44]	Mohamed-Ghazal et al. [45]	Shehata and Hamza [46]	Shehata et al. [47]	Tantawy et al. [48]	Imam et al. [49]	Nammour et al. [50]
1. Was true randomization used for the assignment of participants to treatment groups?	Y	Y	Y	Y	Y	Y	Y	Y
2. Was allocation to treatment groups concealed?	Unclear	Unclear	Unclear	Unclear	Unclear	Unclear	Unclear	Unclear
3. Were treatment groups similar at the baseline?	Y	Y	Y	Y	Y	Y	Y	Y
4. Were participants blind to treatment assignment?	Unclear	Unclear	Unclear	Y	Unclear	Unclear	Unclear	Unclear
5. Were those delivering treatment blind to treatment assignment?	Unclear	Unclear	Unclear	Y	Unclear	Unclear	Unclear	Unclear
6. Were outcomes assessors blind to treatment assignment?	Unclear	Unclear	Unclear	Y	Unclear	Unclear	Unclear	Unclear
7. Were treatment groups treated identically, other than the intervention of interest?	Y	Y	Y	Y	Y	Y	Y	Y
8. Was follow-up complete, and if not, were differences between groups in terms of their follow-up adequately described and analyzed?	Y	Y	Y	Y	Y	Y	Y	Y
9. Were participants analyzed in the groups to which they were randomized?	Y	Y	Y	Y	Y	Y	Y	Y
10. Were outcomes measured in the same way for treatment groups?	Y	Y	Y	Y	Y	Y	Y	Y
11. Were outcomes measured in a reliable way?	Y	Y	Y	Y	Y	Y	Y	Y
12. Was appropriate statistical analysis used?	Y	Y	Y	Y	Y	Y	Y	Y
13. Was the trial design appropriate, and any deviations from the standard RCT design (individual randomization, parallel groups) accounted for in the conduct and analysis of the trial?	Y	Y	Y	Y	Y	Y	Y	Y

Discussion

This systematic review and quantitative synthesis provide an in-depth examination of the patterns, outcomes, and complications associated with PCI across diverse settings in Africa. The analysis of 31 studies from nine countries reveals valuable insights into real-world interventional cardiology practices on the continent, highlighting areas of progress and ongoing challenges in managing CAD.

Geographic and Temporal Trends

The geographical skew in publications, with Egypt accounting for over 60% of included studies, underscores the uneven distribution of interventional cardiology infrastructure across Africa. This pattern has been echoed in prior reviews, which identified North African nations, particularly Egypt and Tunisia, as early adopters of catheterization labs and coronary interventions due to more robust healthcare financing and earlier specialist training programs [51,52]. Sub-Saharan Africa, by contrast, remains underrepresented, reflecting ongoing disparities in access to advanced cardiovascular care [53].

The time frame of the studies (2008-2024) also reveals an encouraging expansion in cardiovascular research capacity over the past decade. However, limitations in long-term follow-up and patient registries remain pervasive, as seen in the 12 studies that failed to report follow-up periods, limiting assessment of longitudinal outcomes.

Demographic and Clinical Profile

The predominance of male patients (74.3%) and the mean age bracket of 53-66 years are consistent with epidemiologic data suggesting earlier onset of ischemic heart disease in African populations, especially among men [54,55]. The clustering of risk factors - including smoking (47.2%), hypertension (46.2%), and diabetes mellitus (41.5%) - further underscores the syndemic interaction between modifiable risk exposures and premature cardiovascular morbidity [56].

Interestingly, these data reaffirm the tobacco epidemic as a leading modifiable risk factor for CAD in Africa, a finding mirrored in recent continental meta-analyses [57]. Despite global declines in smoking prevalence, Sub-Saharan African regions continue to experience rising tobacco use, driven by aggressive marketing and weak regulatory enforcement.

Procedure Practices and Reperfusion Outcomes

Our analysis shows a notable shift toward the radial approach, utilized in 56.8% of cases, compared to 43.2% femoral access. This mirrors global best practices favoring radial access due to reduced bleeding complications and early patient mobilization [58,59]. The adoption of this approach in African settings indicates growing technical proficiency, albeit unevenly distributed.

Furthermore, the LAD was the most commonly affected vessel (60.2%), in keeping with its role as a primary contributor to myocardial infarction severity [60]. Stent-based PCI was the dominant technique, used in 97% of cases, with near-equal distribution of bare-metal and drug-eluting stents. However, the substantial use of bare-metal stents (34.4%) may point to persistent cost barriers limiting access to advanced devices, a challenge also identified by Alhuneafat et al. (2024) in studies of interventional equity [61].

Notably, 90% of all PCIs were performed for STEMI, emphasizing the emergency-driven nature of interventional care on the continent. This is indicative of both delayed presentation and limited availability of elective diagnostic testing, which in high-income countries permits more stable CAD cases to benefit from PCI under controlled conditions [62].

Reperfusion Success and Outcome Metrics

The post-procedural TIMI grade III flow achieved in 78.2% of cases is encouraging and reflects technical success in the majority of PCI procedures. These results are comparable to global averages reported in multicenter registries such as HORIZONS-AMI and GRACE, where a post-procedural TIMI grade III flow of 90% and 89% respectively, was reported [63,64]. Nevertheless, the 4.9% all-cause mortality, with 4.7% in-hospital mortality, remains concerning, albeit not unexpected. The mortality rates are higher than in high-resource countries, where figures range between 1.5% and 2.5% for STEMI PCI, and they reflect the impact of systemic factors such as delays to reperfusion, suboptimal medication adherence, and lack of post-discharge care infrastructure [54,65].

Complications were frequent, particularly heart failure (19.1%), arrhythmia (9.1%), and revascularization needs (8.9%), pointing to substantial post-procedure morbidity. These complications echo the systemic limitations of care continuity, especially in resource-constrained environments where early discharge is often followed by inadequate outpatient follow-up and poor medication access [51]. The high MACE rate (17.8%), although derived from a subset of studies, signals the need for structured secondary prevention programs and better risk stratification tools for African populations.

Implications for Policy and Practice

This review illustrates both advances and persistent gaps in PCI practice across Africa. While technical proficiency and procedural success appear comparable to international standards, inequities in access, device availability, and post-procedural care contribute to suboptimal long-term outcomes. These findings underscore the urgency of adopting continental policy frameworks such as the PASCAR roadmap for noncommunicable diseases, which calls for investments in catheterization labs, workforce training, and universal health coverage [51,61].

Additionally, national health ministries must prioritize cardiovascular registries that systematically collect and report PCI outcomes, thereby enhancing transparency, quality improvement, and clinical benchmarking.

Strengths and limitations

Strengths

This study offers a comprehensive and methodologically robust synthesis of PCI practices and outcomes across diverse African settings - a region historically underrepresented in global cardiovascular literature. A key strength lies in the systematic approach applied to study selection, adhering to PRISMA guidelines, which ensured transparent and reproducible data screening, eligibility, and extraction.

Moreover, by restricting inclusion to original research conducted exclusively in African populations, the study uniquely reflects the regional heterogeneity in demographics, healthcare access, procedural standards, and clinical outcomes. This regional specificity enhances the contextual validity of findings and provides locally - relevant evidence to inform health policy and clinical decision-making across the continent.

In terms of breadth, this review is the most up-to-date and largest synthesis of PCI in Africa to date, including studies from nine countries, with a total patient population of over 11,500, covering a wide temporal span (2008-2024). The inclusion of cohort studies, randomized controlled trials, and cross-sectional studies adds to the robustness of the conclusions and offers both longitudinal and cross-sectional perspectives on PCI performance in the region.

The study also excels in capturing granular procedural details (e.g., vascular access routes, coronary artery involvement, stent type, TIMI flow grades, and complication profiles) and linking them with key clinical outcomes (MACE, mortality, heart failure, etc.), offering a clinically meaningful, data-driven overview of PCI success and gaps in African contexts.

Finally, the use of standardized outcome definitions (e.g., TIMI flow grade, MACE) supports comparability with international studies and registries, enhancing the external validity and relevance of the findings for both regional and global cardiology communities.

Limitations

Despite its strengths, this study has several limitations that must be acknowledged. First, there is a significant geographic imbalance in the included studies, with Egypt alone contributing more than 60% of all eligible research. This overrepresentation likely reflects differential research capacity, funding, and infrastructure across African countries, and may limit the generalizability of findings to underrepresented regions such as Central, West, and parts of East Africa.

Second, many studies had incomplete reporting of key clinical variables. For example, 12 studies lacked follow-up duration, and several did not specify gender distribution, stent type, or complication timelines. Such missing data limit the ability to conduct pooled meta-analyses and may introduce information bias, especially if the omitted data are systematically associated with worse outcomes or underreporting of complications.

Third, the heterogeneity in study design and outcome reporting complicates comparisons across studies. Definitions of MACE, revascularization, and post-PCI complications were not standardized in all cases, which could lead to outcome misclassification and reduced internal consistency.

Fourth, while the study aimed to assess post-procedural outcomes, long-term outcomes (beyond 12 months) were reported in only a small subset of studies. This hinders the evaluation of stent durability, late thrombotic events, and the effectiveness of secondary prevention strategies-areas that are critical in guiding best practice and policy implementation.

Fifth, the review excluded non-English articles, which may have led to the omission of relevant French-language or Arabic-language research from countries like Algeria, Morocco, or parts of West and Central Africa, where French is the predominant academic language.

Lastly, as this is a review of observational studies, the findings are susceptible to selection bias, confounding, and publication bias. Most included studies lacked randomization, and very few adjusted for baseline covariates, comorbidities, or pre-hospital delays, which are known to impact PCI outcomes significantly.

Recommendations and future research

The findings of this study underscore the substantial progress made in interventional cardiology across parts of Africa, while also revealing critical disparities in access, data quality, procedural uniformity, and long-term patient follow-up. To build upon this emerging foundation, the following multi-level recommendations are proposed to guide clinical practice, health policy, and future research efforts:

1. Establishment of Regional and Continental PCI Registries: There is a pressing need for the creation and implementation of prospective, multicenter registries dedicated to capturing data on PCI procedures, outcomes, complications, and long-term survival across Africa. These registries should adopt standardized reporting frameworks such as the Utstein template or VARC definitions and should be integrated within existing health information systems. Such efforts would mirror initiatives like the GRACE and EUROASPIRE registries, which have significantly improved cardiovascular care benchmarking globally [64,66].

2. Improve Equitable Access to PCI Infrastructure and Devices: The heavy concentration of PCI procedures in North Africa reflects stark inequities in cardiovascular infrastructure across the continent. Governments and development partners must prioritize decentralizing catheterization services through strategic investments in cardiac catheterization labs, particularly in the underserved West, Central, and East African regions. Public-private partnerships could facilitate the procurement of cost-effective stents (especially drug-eluting stents), and local manufacturing initiatives could reduce reliance on imported consumables.

3. Strengthen Human Resource Capacity: Expanding the training and accreditation of interventional cardiologists, catheterization lab nurses, and cardiovascular technologists is essential. Pan-African academic collaborations, fellowships, and South-South knowledge exchanges (e.g., between Egypt and sub-Saharan institutions) should be fostered. Additionally, integrating PCI competencies into national cardiovascular training curricula will create a more sustainable workforce pipeline.

4. Promote Guideline-Concordant, Risk-Stratified PCI Care: Standardizing clinical decision-making through context-specific adaptations of international PCI guidelines (e.g., ESC/EACTS, ACC/AHA) is essential. This includes promoting risk stratification tools for patient selection (e.g., SYNTAX, GRACE scores), establishing protocols for early invasive strategies in STEMI, and reinforcing post-procedure management with dual antiplatelet therapy (DAPT), statins, and cardiac rehabilitation.

These protocols must be adapted to local realities-e.g., low insurance coverage, rural-urban gaps-and integrated into national health policies.

5. Enhance Post-Procedure Surveillance and Continuity of Care: There is a clear gap in post-discharge surveillance and long-term patient follow-up, as evidenced by the lack of follow-up data in several studies. Future studies should evaluate medication adherence and risk-factor control, late stent thrombosis and restenosis rates and quality of life and functional status post-PCI.

Digital health platforms and mobile-based follow-up systems may offer innovative, scalable solutions for continuity of care in low-resource settings.

6. Prioritize Under-Researched Populations and Conditions: Future research should address gender disparities, as the current evidence base is male-dominated. Additionally, special attention is needed for Younger patients, in whom early CAD appears more prevalent in Africa, patients with multiple comorbidities, including HIV and sickle cell disease and rural populations, often excluded from urban hospital-based studies.

Moreover, cost-effectiveness analyses of PCI versus medical therapy in African settings are critically needed to guide national financing decisions.

## Conclusions

With the rising use of PCI on the continent, this study serves to fill a major research gap by providing comprehensive data on clinical outcomes associated with this intervention in Africa. The findings highlight that while procedural success, reflected in high rates of stent deployment and post-procedure TIMI grade III flow, is encouraging and comparable to global benchmarks, substantial disparities persist in access, procedural practices, and post-PCI outcomes across the continent. STEMI remains the predominant indication for PCI, underscoring a healthcare system that is still largely reactive rather than preventive. The high burden of comorbidities such as smoking, hypertension, and diabetes, paired with complication rates including heart failure and MACE, reflects systemic gaps in both acute and chronic cardiovascular care. Marked geographic imbalances, incomplete data reporting, and limited long-term follow-up constrain the generalizability of findings and underscore the urgent need for investment in national and regional PCI registries, equitable expansion of interventional cardiology infrastructure, and integration of PCI data into broader noncommunicable disease surveillance frameworks.

To improve outcomes and close the equity gap, African health systems must prioritize context-sensitive guideline implementation, capacity building, and continuity of care. Future research should focus on prospective multicenter studies, cost-effectiveness analysis, and underrepresented populations to inform a more inclusive and sustainable cardiology practice across the continent.

## Appendices

**Table 9:** Search Strategy (string) AJOL: African Journals Online

No	Database	Keyword Search	No of Articles
1	PubMed	PCI AND AFRICA ("Percutaneous Coronary Intervention"[MeSH Terms] OR ("percutaneous"[All Fields] AND "coronary"[All Fields] AND "intervention"[All Fields]) OR "Percutaneous Coronary Intervention"[All Fields] OR ("Percutaneous Coronary Intervention"[All Fields] OR "PCI"[All Fields] OR "Coronary reperfusion"[All Fields] OR "Coronary angioplasty"[All Fields] OR "Coronary revascularization"[All Fields] OR "drug eluting stent"[All Fields] OR "stent"[All Fields] OR "Angioplasty"[All Fields] OR "drug-eluting balloon"[All Fields] OR "percutaneous transluminal coronary angioplasty"[All Fields])) AND ("Africa"[MeSH Terms] OR ("Africa"[MeSH Terms] OR "Africa"[All Fields] OR "africa s"[All Fields] OR "africas"[All Fields]) OR (((("Africa"[All Fields] OR "African"[All Fields] OR "Algeria"[All Fields] OR "Angola"[All Fields] OR "Benin"[All Fields] OR "Botswana"[All Fields] OR "Burkina Faso"[All Fields] OR "Burundi"[All Fields] OR "Cabo Verde"[All Fields] OR "Cameroon"[All Fields] OR "Central African Republic"[All Fields] OR "Chad"[All Fields] OR "Congo"[All Fields] OR "Cote d'Ivoire"[All Fields] OR "Democratic Republic of the Congo"[All Fields] OR "Djibouti"[All Fields] OR "Egypt"[All Fields] OR "Equatorial Guinea"[All Fields] OR "Eritrea"[All Fields] OR "Eswatini"[All Fields] OR "Ethiopia"[All Fields] OR "Gabon"[All Fields] OR "Gambia"[All Fields] OR "Ghana"[All Fields] OR "Guinea"[All Fields] OR "Guinea-Bissau"[All Fields] OR "Kenya"[All Fields] OR "Lesotho"[All Fields] OR "Liberia"[All Fields] OR "Libya"[All Fields] OR "Malawi"[All Fields] OR "Mali"[All Fields] OR "Mauritania"[All Fields] OR "Morocco"[All Fields] OR "Mozambique"[All Fields] OR "Namibia"[All Fields]) AND "Or"[All Fields]) AND "Niger"[All Fields]) OR "Nigeria"[All Fields] OR "Rwanda"[All Fields] OR "Sao Tome and Principe"[All Fields] OR "Senegal"[All Fields] OR "Sierra Leone"[All Fields] OR "Somalia"[All Fields] OR "South Africa"[All Fields] OR "South Sudan"[All Fields] OR "Sudan"[All Fields] OR "Tanzania"[All Fields] OR "Togo"[All Fields] OR "Tunisia"[All Fields] OR "Uganda"[All Fields] OR "Zambia"[All Fields] OR "Zimbabwe"[All Fields]))	687
		PCI AND TREATMENT OUTCOME AND AFRICA ("Percutaneous Coronary Intervention"[MeSH Terms] OR ("percutaneous"[All Fields] AND "coronary"[All Fields] AND "intervention"[All Fields]) OR "Percutaneous Coronary Intervention"[All Fields] OR ("Percutaneous Coronary Intervention"[All Fields] OR "PCI"[All Fields] OR "Coronary reperfusion"[All Fields] OR "Coronary angioplasty"[All Fields] OR "Coronary revascularization"[All Fields] OR "drug eluting stent"[All Fields] OR "stent"[All Fields] OR "Angioplasty"[All Fields] OR "drug-eluting balloon"[All Fields] OR "percutaneous transluminal coronary angioplasty"[All Fields])) AND ("Treatment Outcome"[MeSH Terms] OR ("Outcome"[All Fields] OR "Treatment Outcome"[All Fields] OR "Outcomes"[All Fields] OR "Mortality"[All Fields] OR "major adverse cardiovascular event"[All Fields] OR "MACE"[All Fields] OR "Complication"[All Fields] OR "Length of hospital stay"[All Fields] OR "Success rate"[All Fields])) AND ("Africa"[MeSH Terms] OR ("Africa"[MeSH Terms] OR "Africa"[All Fields] OR "africa s"[All Fields] OR "africas"[All Fields]) OR (((("Africa"[All Fields] OR "African"[All Fields] OR "Algeria"[All Fields] OR "Angola"[All Fields] OR "Benin"[All Fields] OR "Botswana"[All Fields] OR "Burkina Faso"[All Fields] OR "Burundi"[All Fields] OR "Cabo Verde"[All Fields] OR "Cameroon"[All Fields] OR "Central African Republic"[All Fields] OR "Chad"[All Fields] OR "Congo"[All Fields] OR "Cote d'Ivoire"[All Fields] OR "Democratic Republic of the Congo"[All Fields] OR "Djibouti"[All Fields] OR "Egypt"[All Fields] OR "Equatorial Guinea"[All Fields] OR "Eritrea"[All Fields] OR "Eswatini"[All Fields] OR "Ethiopia"[All Fields] OR "Gabon"[All Fields] OR "Gambia"[All Fields] OR "Ghana"[All Fields] OR "Guinea"[All Fields] OR "Guinea-Bissau"[All Fields] OR "Kenya"[All Fields] OR "Lesotho"[All Fields] OR "Liberia"[All Fields] OR "Libya"[All Fields] OR "Malawi"[All Fields] OR "Mali"[All Fields] OR "Mauritania"[All Fields] OR "Morocco"[All Fields] OR "Mozambique"[All Fields] OR "Namibia"[All Fields]) AND "Or"[All Fields]) AND "Niger"[All Fields]) OR "Nigeria"[All Fields] OR "Rwanda"[All Fields] OR "Sao Tome and Principe"[All Fields] OR "Senegal"[All Fields] OR "Sierra Leone"[All Fields] OR "Somalia"[All Fields] OR "South Africa"[All Fields] OR "South Sudan"[All Fields] OR "Sudan"[All Fields] OR "Tanzania"[All Fields] OR "Togo"[All Fields] OR "Tunisia"[All Fields] OR "Uganda"[All Fields] OR "Zambia"[All Fields] OR "Zimbabwe"[All Fields]))	438
		PCI AND COMPLICATIONS AND AFRICA ("Percutaneous Coronary Intervention"[MeSH Terms] OR ("percutaneous"[All Fields] AND "coronary"[All Fields] AND "intervention"[All Fields]) OR "Percutaneous Coronary Intervention"[All Fields] OR ("Percutaneous Coronary Intervention"[All Fields] OR "PCI"[All Fields] OR "Coronary reperfusion"[All Fields] OR "Coronary angioplasty"[All Fields] OR "Coronary revascularization"[All Fields] OR "drug eluting stent"[All Fields] OR "stent"[All Fields] OR "Angioplasty"[All Fields] OR "drug-eluting balloon"[All Fields] OR "percutaneous transluminal coronary angioplasty"[All Fields])) AND ("complicances"[All Fields] OR "complicate"[All Fields] OR "complicated"[All Fields] OR "complicates"[All Fields] OR "complicating"[All Fields] OR "complication"[All Fields] OR "complication s"[All Fields] OR "complications"[MeSH Subheading] OR "complications"[All Fields]) AND ("Africa"[MeSH Terms] OR ("Africa"[MeSH Terms] OR "Africa"[All Fields] OR "africa s"[All Fields] OR "africas"[All Fields]) OR (((("Africa"[All Fields] OR "African"[All Fields] OR "Algeria"[All Fields] OR "Angola"[All Fields] OR "Benin"[All Fields] OR "Botswana"[All Fields] OR "Burkina Faso"[All Fields] OR "Burundi"[All Fields] OR "Cabo Verde"[All Fields] OR "Cameroon"[All Fields] OR "Central African Republic"[All Fields] OR "Chad"[All Fields] OR "Congo"[All Fields] OR "Cote d'Ivoire"[All Fields] OR "Democratic Republic of the Congo"[All Fields] OR "Djibouti"[All Fields] OR "Egypt"[All Fields] OR "Equatorial Guinea"[All Fields] OR "Eritrea"[All Fields] OR "Eswatini"[All Fields] OR "Ethiopia"[All Fields] OR "Gabon"[All Fields] OR "Gambia"[All Fields] OR "Ghana"[All Fields] OR "Guinea"[All Fields] OR "Guinea-Bissau"[All Fields] OR "Kenya"[All Fields] OR "Lesotho"[All Fields] OR "Liberia"[All Fields] OR "Libya"[All Fields] OR "Malawi"[All Fields] OR "Mali"[All Fields] OR "Mauritania"[All Fields] OR "Morocco"[All Fields] OR "Mozambique"[All Fields] OR "Namibia"[All Fields]) AND "Or"[All Fields]) AND "Niger"[All Fields]) OR "Nigeria"[All Fields] OR "Rwanda"[All Fields] OR "Sao Tome and Principe"[All Fields] OR "Senegal"[All Fields] OR "Sierra Leone"[All Fields] OR "Somalia"[All Fields] OR "South Africa"[All Fields] OR "South Sudan"[All Fields] OR "Sudan"[All Fields] OR "Tanzania"[All Fields] OR "Togo"[All Fields] OR "Tunisia"[All Fields] OR "Uganda"[All Fields] OR "Zambia"[All Fields] OR "Zimbabwe"[All Fields]))	282
2	EMBASE	PCI AND AFRICA (("Percutaneous Coronary Intervention" or "PCI" or "Coronary reperfusion" or "Coronary angioplasty" or "Coronary revascularization" or "drug eluting stent" or "stent" or "Angioplasty" or "drug-eluting balloon" or "percutaneous transluminal coronary angioplasty") and Africa).af.	1509
		PCI AND TREATMENT OUTCOME AND AFRICA (("Percutaneous Coronary Intervention" or "PCI" or "Coronary reperfusion" or "Coronary angioplasty" or "Coronary revascularization" or "drug eluting stent" or "stent" or "Angioplasty" or "drug-eluting balloon" or "percutaneous transluminal coronary angioplasty") and ("Outcome" or "Treatment Outcome" or "Outcomes" or "Mortality" or "major adverse cardiovascular event" or "MACE" or "Complications" or "Length of hospital stay" or "Success rate") and Africa).af.	902
		PCI AND COMPLICATIONS AND AFRICA (("Percutaneous Coronary Intervention" or "PCI" or "Coronary reperfusion" or "Coronary angioplasty" or "Coronary revascularization" or "drug eluting stent" or "stent" or "Angioplasty" or "drug-eluting balloon" or "percutaneous transluminal coronary angioplasty") and Complication and Africa).af.	485
3	AJOL (10 pages)	Percutaneous coronary intervention OR PCI	100
